# Mediating kinase activity in Ras-mutant cancer: potential for an individualised approach?

**DOI:** 10.3389/fphar.2024.1441938

**Published:** 2024-09-20

**Authors:** Fiona M. Healy, Amy L. Turner, Vanessa Marensi, David J. MacEwan

**Affiliations:** ^1^ Department of Pharmacology and Therapeutics, Institute of Systems, Molecular and Integrative Biology, University of Liverpool, Liverpool, United Kingdom; ^2^ Department of Biochemistry, Cell and Systems Biology, Institute of Systems, Molecular and Integrative Biology, University of Liverpool, Liverpool, United Kingdom; ^3^ Chester Medical School, University of Chester, Chester, United Kingdom

**Keywords:** cancer, kinase, mutation, Ras, signalling, targeted therapy

## Abstract

It is widely acknowledged that there is a considerable number of oncogenic mutations within the Ras superfamily of small GTPases which are the driving force behind a multitude of cancers. Ras proteins mediate a plethora of kinase pathways, including the MAPK, PI3K, and Ral pathways. Since Ras was considered undruggable until recently, pharmacological targeting of pathways downstream of Ras has been attempted to varying success, though drug resistance has often proven an issue. Nuances between kinase pathway activation in the presence of various Ras mutants are thought to contribute to the resistance, however, the reasoning behind activation of different pathways in different Ras mutational contexts is yet to be fully elucidated. Indeed, such disparities often depend on cancer type and disease progression. However, we are in a revolutionary age of Ras mutant targeted therapy, with direct-targeting KRAS-G12C inhibitors revolutionising the field and achieving FDA-approval in recent years. However, these are only beneficial in a subset of patients. Approximately 90% of Ras-mutant cancers are not KRAS-G12C mutant, and therefore raises the question as to whether other distinct amino acid substitutions within Ras may one day be targetable in a similar manner, and indeed whether better understanding of the downstream pathways these various mutants activate could further improve therapy. Here, we discuss the favouring of kinase pathways across an array of Ras-mutant oncogenic contexts and assess recent advances in pharmacological targeting of various Ras mutants. Ultimately, we will examine the utility of individualised pharmacological approaches to Ras-mediated cancer.

## Introduction

The Ras superfamily of small guanosine triphosphate hydrolases (GTPases) underpins cell signalling, in both health and disease (Simanshu et al., 2017). Though not a kinase itself, GTP-bound (active) Ras has the power to activate a multitude of downstream kinases, which control multiple cellular mechanisms and maintain intracellular functions in a homeostatic manner (Simanshu et al., 2017).

There are three key Ras isoforms, namely NRAS, HRAS and KRAS. In addition, there are two splice variants occurring in exon 4 of *KRAS*, thereby rendering variants KRAS-4A and KRAS-4B (Prior et al., 2012). KRAS-4B has been shown to be most expressed across a range of cancer cell lines and healthy mouse tissue, followed by KRAS-4A, NRAS and lastly HRAS (Hood et al., 2023). Ras signalling is essential in a myriad of tissues, and therefore each Ras isoform is ubiquitously expressed in the body. However, expression of each Ras isoform is highest in the gastrointestinal tract, with considerable expression also seen in the blood system, immune system, reproductive system and brain (The Human Protein Atlas, 2023; Uhlén et al., 2015). Sampling of mouse tissues showed differential expression of Ras isoforms in neonates and adults, suggesting that expression of different isoforms fluctuates during development. Broadly speaking however, KRas is most expressed, followed by NRas and then HRas (Newlaczyl et al., 2017). This is in line with Ras being known as a key regulator of proliferative, survival and differentiation pathways, across various tissues.

**FIGURE 1:**
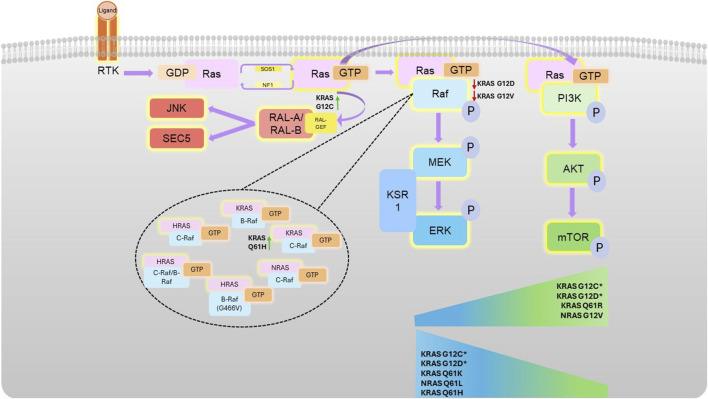
Ras-mediated kinase pathways. Following receptor tyrosine kinase (RTK) activation, guanine nucleotide exchange factors activate Ras by facilitating a switch in binding of GDP for GTP. Activated Ras can associate with Raf (which homo- or hetero-dimerises and activates), or PI3K, both at the effector lobe of Ras. Activation of these kinases then stimulates subsequent downstream activation (phosphorylation) the MAPK or AKT pathway, respectively. Different Ras mutations confer different propensities for Raf isoform binding (as shown in the top of the figure), with certain mutations conferring preference for certain pathways, illustrated by the sliding scales. G12C and G12D pathway preference is highly context dependent, as denoted by * and detailed in the text.

There are three key structural regions of Ras, namely the effector region, which includes the P loop and switch regions, allosteric region and hypervariable region (HVR) (Hobbs et al., 2016) (Figure 1). As the name suggests, the effector region is where Ras effector molecules bind, such as the Ral GTPase-activating protein (Ral-GAP) and the serine/threonine-protein Raf kinases (Tran et al., 2021). This region, which spans approximately half of the total Ras protein, is fully conserved throughout KRAS, NRAS, and HRAS (Healy et al., 2022; Prior et al., 2012). The allosteric region comprises the next ∼80 amino acids and exhibits a high degree of homology between each of the isoforms and is involved in Ras association at the cell membrane, as well as likely playing a role in conferring isoform-specific signalling differences (Prior et al., 2012; Gorfe et al., 2007). The HVR is required for Ras trafficking to the cell membrane, it comprises the farnesylation site and presents little sequence fidelity between the different isoforms (Healy et al., 2022; Prior et al., 2012) (Figure 1).

Ras proteins transition between intracellular compartments to regulate cell signalling (Prior and Hancock, 2012). This process is largely regulated by direct interaction with different intracellular membranes. KRAS, NRAS, and HRAS are all *S*-prenylated by a farnesyl group at the *C*-terminal cysteine in the CAAX sequence motif within the hypervariable region, which provides a signal cleavage for the endoproteolytic enzymes, Ras Converting CAAX Endopeptidase 1 (Rce1) to cleave the AAX, exposing the *C*-terminal cysteine to other post-translational modifications (Chen et al., 1996; Hildebrandt et al., 2024; Michaelson et al., 2005). *In vitro* studies on KRas-4B show that the isoprenylated Ras can be further methylated at the carboxyl-terminal isoprenylcysteine by isoprenylcysteine carboxyl methyltransferase (Icmt) (Bergo et al., 2000). These structural changes provide increased affinity to cellular membranes, controlling its trafficking from the Golgi to the plasma membrane. At the membrane, a polybasic amino acid sequence increases affinity with the anionic phospholipids in the membrane to stabilize KRAS at the site (Hancock et al., 1990).

When EGFP was engineered with the C-terminus of HRAS (*C*MS*C*K*C*VLS) or KRAS (*KKKKKK*SKTK*C*VIM), with the membrane binding domain, it caused differential localization of EGFP to the membrane. The EGFP with an HRAS extension was partitioned between intracellular and plasma membranes. Meanwhile, the EGFP containing a KRAS extension was found to immediately localized to the plasma membrane, as result of the polybasic domain. Lysine substitution to the negatively charged glutamine prevented interaction with the plasma membrane, demonstrating that farnesylation of the C-terminus solely is not sufficient for stable localization at the plasma membrane (Apolloni et al., 2000). Therefore, a polybasic domain allows persistent localization of KRAS to the membrane whereas reversible palmitoylation regulates the turnover of HRAS proteins to form internal membranes to the plasma membrane. NRAS and HRAS do not contain a polybasic sequence, they are further lipidated at a neighbouring cysteine residue (Javanainen et al., 2017; Simons and Toomre, 2000; Simons and Ikonen, 1997) bringing Ras proximity to the membrane facilitates complexation with Son of Sevenless (SOS) and kinase receptors to initiate downstream activity (Christensen et al., 2016) suggesting that dynamic regulation of Ras on-an-off from the membrane is critical to switch the signalling cascade.

Inactive HRAS was shown to interact with caveolin, a protein present in cholesterol-rich regions required for clathrin-independent internalization of several receptors in the cell surface (Li et al., 1996; Song et al., 1996). Likewise, the SRC proto-oncogene non-receptor kinase, G protein α subunits and other downstream effectors, including Raf (Jaumot et al., 2002; Li et al., 1996). This suggests HRAS can interact with these proteins in cholesterol-rich regions to promote signalling.

Ras proteins are small GTPases, active when in its GTP-bound state, and inactive when GDP is bound. In physiological situations, Ras isoforms cycle between these two states, thereby exercising control over activation of downstream kinases (Killoran and Smith, 2019; Milburn et al., 1990). Ras can be activated by Guanine nucleotide Exchange Factors (GEFs), to facilitate its conversion from the inactive GDP-bound state to the active GTP-bound state, and it is this GTP-bound state which is necessary to facilitate the activation of downstream kinases. One such GEF which plays a critical role is Son of Sevenless homolog 1 (SOS1). SOS1 binds Ras at the P loop, Switch I and Switch II regions within the effector lobe (Figure 1), facilitating an open conformation of the nucleotide binding domain, restricting magnesium and phosphate binding to this region of Ras (as in its inactive conformation) and instead permitting GTP to bind and activate Ras (Boriack-Sjodin et al., 1998). Different Ras isoforms are believed to be activated and de-activated by different GEFs and GTPase Activating Proteins (GAPs), respectively. For example, Ras-specific guanine-nucleotide exchange factor (Ras-GRF) is believed to activate HRas only (Jones and Jackson, 1998). SOS2, of the same GEF family as SOS1, displays a hierarchy for binding to the Ras isoforms, with KRAS being most reliant of the three isoforms on SOS2 to drive oncogenesis, followed by NRAS and lastly HRAS (Sheffels et al., 2018). Conversely, neurofibromin-1 GAP (NF1-GAP) has been previously shown have four-fold higher binding affinity to HRAS, compared to NRAS (Bollag and McCormick, 1991).

However, when Ras isoforms are mutated, this process becomes dysregulated, and the physiological Ras-GDP/Ras-GTP equilibrium becomes unbalanced leading to the development of diseases (Haigis et al., 2008; Hobbs et al., 2016; Killoran and Smith, 2019). Common mutations within each of the Ras isoforms are typically considered to cause an imbalance in the cycling of Ras between its GTP-bound (active) state, and its GDP-bound (inactive state). Instead, mutated Ras favours its GTP-bound state, ultimately causing an increase in cell proliferation, dysregulated differentiation and a pro-survival effect (Killoran and Smith, 2019). Ras mutations are often considered oncogenic, and drivers of numerous types of cancers, as well as other conditions. Key Ras-driven cancers include pancreatic cancer and non-small cell lung cancer (NSCLC), both of which are primarily caused by mutations in KRAS (Cox et al., 2014). Additionally, non-cancerous disorders developed from disturbances in the Ras/MAPK pathway activity are often collectively referred as Rasopathies, which include Noonan syndrome and Costello syndrome (Rauen, 2022). These genetic syndromes are caused by germline mutations in Ras isoforms or in Ras/Mitogen-activated protein kinase (MAPK) pathway genes, inducing aberrant activation of downstream pathways, which may cause developmental issues at an embryonic stage, or post-natal (Tidyman and Rauen, 2009).

**FIGURE 2:**
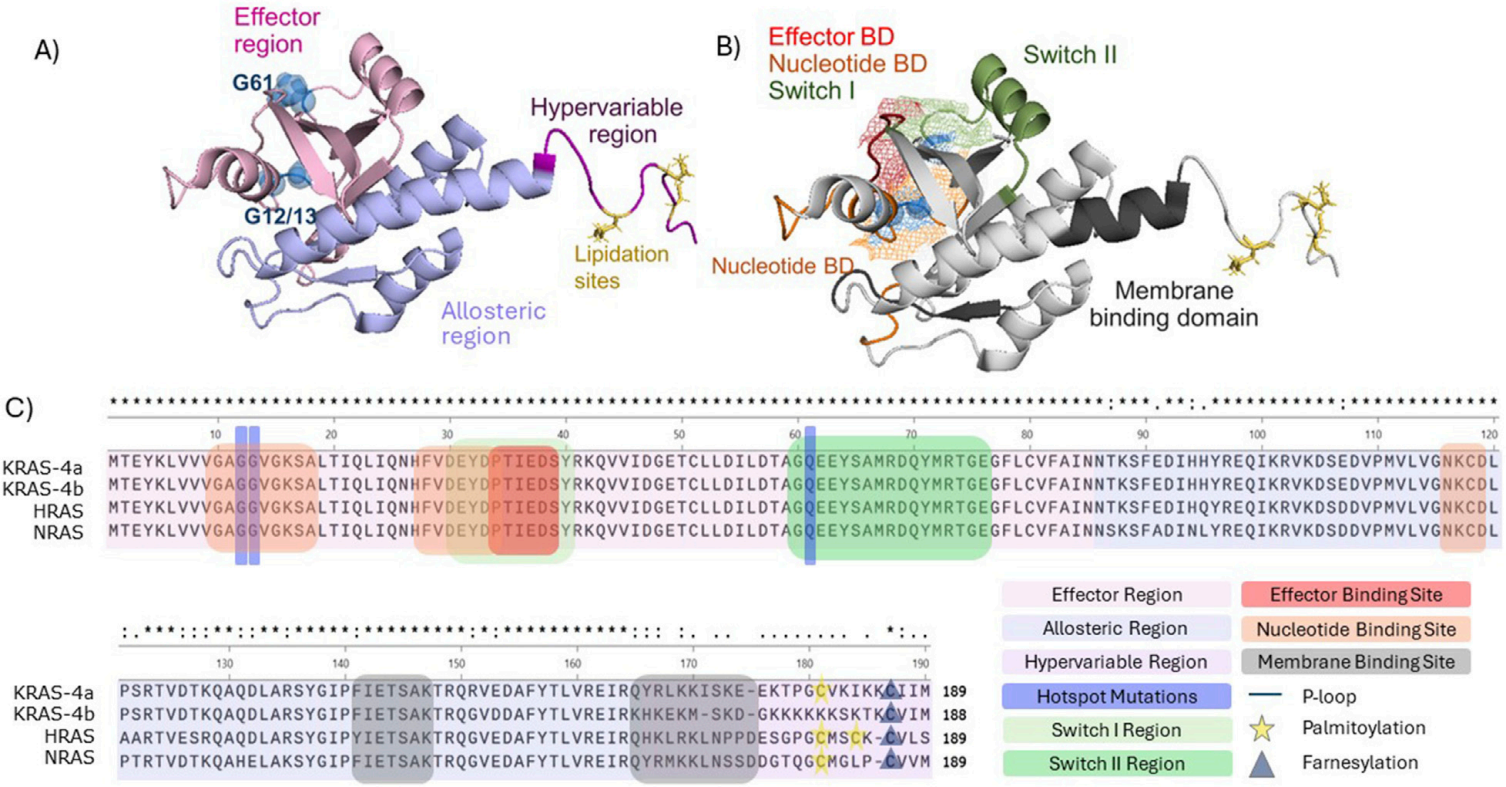
Structural and functional domains of full length KRAS (PBD 7KYZ). **(A)** Colour-coded RAS regions. Effector region (light pink), hypervariable region (purple), allosteric region (light blue), frequently occurring mutations G12, G13, and G61 (blue spheres), lipidation sites (yellow). **(B)** Visualisation of Ras domains. Ras nucleotide domain (orange), effector domain (red), switch domain I (light green), switch II (green), membrane binding domain (dark grey). G12, G13, G61 and lipid domain were labelled as described in **(A)**. Mesh representation of the overlapping switch I, effector and nucleotide domain. Structures were visualized in PyMOL **(C)** Primary structure alignment (Clustal Omega) of the human RAS isoforms in and identification of the functional domains and regions.

Ras primarily regulates the MAPK and Phosphoinositide 3-kinases (PI3K) pathways, which can be both highly associated with oncogenicity (Herrmann et al., 1995; Rodriguez-Viciana et al., 1994) (Figure 2). The Ral pathway is also activated by Ras, which may also lead to tumorigenesis in some cancers, such as pancreatic and colorectal cancers. In contrast to the increased kinase activity of Ras effectors in the MAPK and PI3K pathway in response to their activation by Ras, Ral is a small GTPase activated by RalGEFs, which themselves can be activated in response to active Ras. Ral plays many biological roles in response to its activation by Ras, which often leads to proliferation, however, the response will vary according to the mutational status of Ras (Guin and Theodorescu, 2015; Bodemann and White, 2008).

Key Ras effectors include PI3K, Raf, and Ral (Aksamitiene et al., 2012). Ras permits PI3K accumulation at the cell membrane, enabling its association with downstream effectors (Zhang et al., 2019a). Class I PI3Ks are known to bind Ras. This class consists of α, β, δ, and γ subunits, of which heterodimers result in the generation of the catalytic subunit containing the kinase domain, p110, as well as the regulatory subunit, p85. KRAS, NRAS and HRAS specifically bind and activate the p110α and γ PI3K subunits (Krygowska and Castellano, 2018; Rodriguez-Viciana et al., 2004). The roles of PI3K isoforms differ between physiological and disease contexts, with the p110α subunit important in conferring the KRAS-G12D oncogenic effects in myeloid leukaemia, yet exhibiting less importance in physiological haematopoiesis, as determined by normal haematopoiesis occurring in p110α depleted mice, and increased survival of KRas G12D myeloid leukaemia mouse models when p110α was depleted (Gritsman et al., 2014). Nevertheless, reliance on the p110α isoform is not always necessary in mediating oncogenic effects. KRAS G12R exhibits a considerably decreased affinity for binding to the GEF SOS1, thereby reducing its activating effects on PI3K pathway activity, compared to more commonly considered mutations such as G12V or G12D. However, although PI3K-AKT signalling is reduced in KRAS G12R-mutant pancreatic cancer, it is not fully eradicated, and instead PI3K is thought to act independently to KRAS G12R, through activity of the γ subunit, rather than p110α (Hobbs et al., 2020). Activation of PI3K in a Ras-mutant context is also thought to depend on upstream Receptor Tyrosine Kinase (RTK) signalling. For example, activation of the IGFR1R has been shown to be essential in KRAS-mutant-mediated PI3K activation in Non-Small Cell Lung Cancer (NSCLC) and colorectal cancer (CRC), whereas EGFR appears to have strong importance in KRAS-WT-mediated PI3K activation in NSCLC models (Molina-Arcas et al., 2013; Ebi et al., 2011).

Ordinarily, Ras-Raf interactions promote either homo- or hetero-dimerization of Raf isoforms using the cysteine-rich region of the Ras Binding Domain (RBD) on Raf (Tran et al., 2021). Different Raf isoforms exhibit differing binding capabilities for various Ras isoforms: while C-Raf binds all Ras isoforms, with greatest affinity for KRAS, followed by NRAS and lastly for HRAS, B-Raf exhibits selectivity for KRAS only (Terrell et al., 2019) This selectivity is largely due to the variation in the HVR of each Ras isoform, with the positively-charged polybasic region of KRAS making it particularly amenable to interaction with the acidic N-terminal region of B-Raf (Terrell et al., 2019). In particular, the presence of Leucine at position 89 in B-Raf can affect binding interactions, since C-Raf R89L amino acid substitution prevents interaction with Ras (Fabian et al., 1994). However, heterodimerization of B-Raf and C-Raf induces B-Raf interaction with HRAS, which is essential for B-Raf-mediated downstream signalling, unless B-Raf is mutated itself, such as through the G466V mutation (Terrell et al., 2019). Furthermore, different Raf isoforms exhibit different propensities for conferring the oncogenic properties of mutant KRAS, with C-Raf essential for the oncogenic signalling associated with KRas G12V mutations in Non-Small Cell Lung Cancer (NSCLC) mouse models, and indeed in KRAS G12S, G12C, and G12V-mutant cell lines (Blasco et al., 2011).

Ral is a small GTPase, within the Ras family, and is similarly activated and de-activated using GEFs and GAPs, commonly referred to as RalGEFs and RalGAPs, amongst others. There are two key isoforms of Ral, RalA and RalB, which can act as mediators of downstream Ras signalling and facilitate cross-talk with other Ras-mediated pathways, including PI3K (Martin et al., 2014). This is through interaction at the C-terminal of the Ral Guanine Nucleotide Dissociation Simulator (RalGDS), which binds GTP-bound Ras (Hofer et al., 1994; Spaargaren and Bischoff, 1994).

The Ras/Ral pathway is less kinase-dependent than other Ras effector pathways, although there has previously been discussions of cross-talk between the Ral and PI3K pathway, through activation of mTOR in response to RalB activity (Martin et al., 2014). Such cross-talk resulted in control of numerous processes, including pancreatic and colorectal cancer tumorigenesis, which was reliant on RalGAP-mediated activation of mTOR (Martin et al., 2014).

## Survival outcomes in Ras-mutant cancer

**FIGURE 3:**
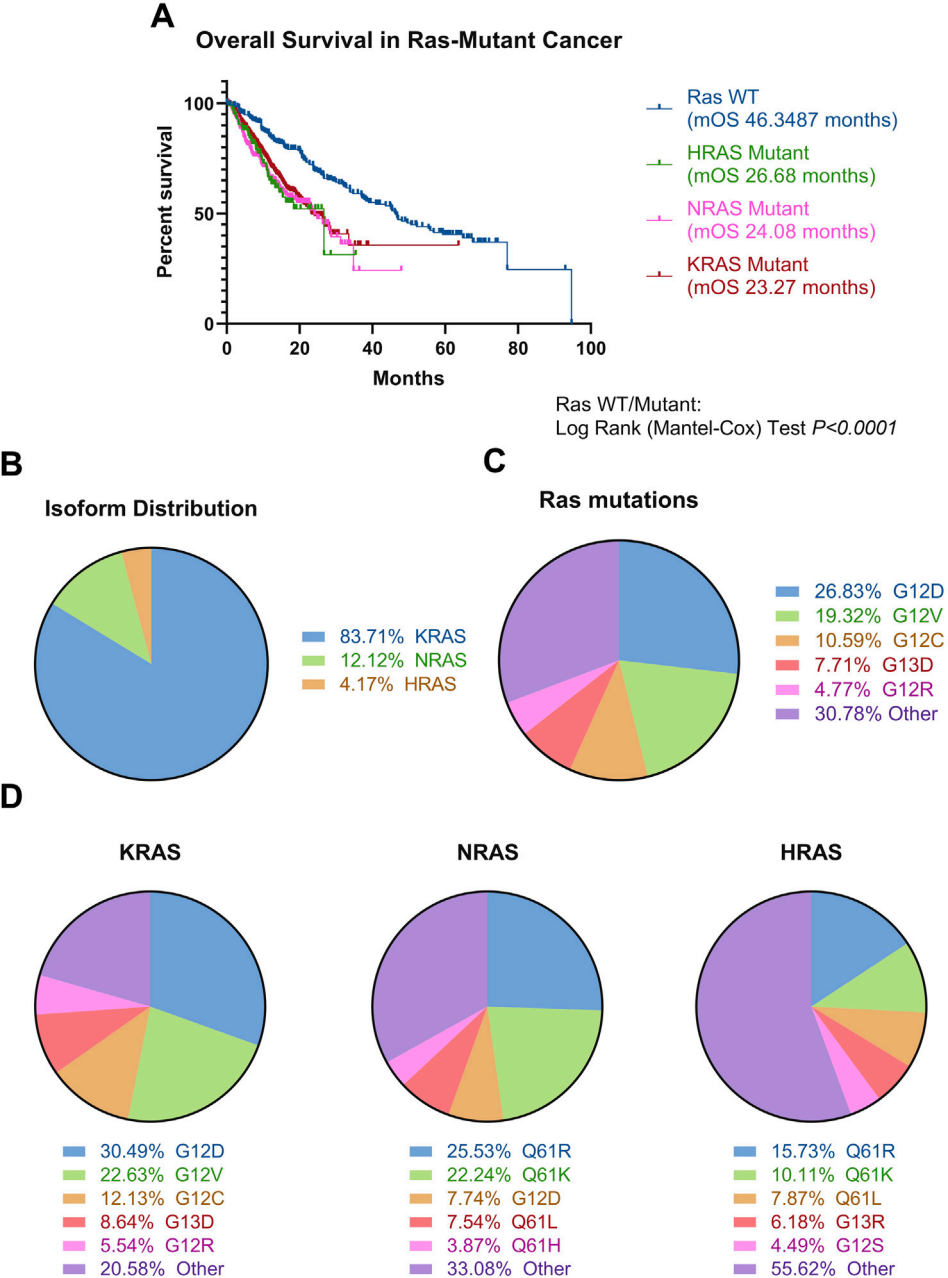
Ras mutations in cancer. **(A)** Ras-mutated patients, stratified by mutated Ras isoform (KRAS N = 3,752, NRAS N = 517, HRAS N = 178), experience poorer overall survival compared to Ras wild-type patients (N = 6,379) (Log-Rank test, P < 0.0001). **(B)** KRAS is the most mutated isoform of the three. N = 24,001 **(C)** There is extreme heterogeneity in the Ras mutational landscape, with over 200 different mutations occurring in Ras isoforms, with these most commonly being at positions G12, G13, and Q61. The top five mutations occurring across all isoforms are denoted in the figure. N = 44,451. This is supported by Supplementary Table S1. **(D)** Mutations stratified by Ras isoform, with top five mutations shown per isoform. Different isoforms have different likelihoods for certain mutations occurring, with HRAS showing the most diversity. The top five most common mutations per isoform denoted on the figure. N = 44,451. This is supported by Supplementary Table S1. Data was analysed from six non-redundant, pan-cancer datasets, obtained through cBioPortal and analysed using GraphPad Prism V8.0.1 and Microsoft Excel (Cerami et al., 2012; Gao et al., 2013; Hyman et al., 2018; ICGC/TCGA Pan-Cancer Analysis of Whole Genomes Consortium, 2020; Miao et al., 2018; Robinson et al., 2017; Wu et al., 2022; Zehir et al., 2017).

Data analysis was conducted for this review from six pan-cancer, non-redundant publicly available databases to study Ras mutational frequency in a pan-cancer context. The datasets analysed here comprised the following: MSK-IMPACT Clinical Sequencing Cohort (MSK, Nat Med 2017), Metastatic Solid Cancers (UMich, Nature 2017), MSS Mixed Solid Tumors (Broad/Dana-Farber, Nat Genet 2018), SUMMIT - Neratinib Basket Study (Multi-Institute, Nature 2018), China Pan-cancer (OrigiMed, Nature 2022) and Pan-cancer analysis of whole genomes (ICGC/TCGA, Nature 2020). Survival and mutation data was extracted from cBioPortal, with Ras mutation frequencies and Kaplan-Meier curves calculated using Microsoft Excel and GraphPad Prism (Figure 3) (Cerami et al., 2012; Gao et al., 2013; Hyman et al., 2018; Miao et al., 2018; ICGC/TCGA Pan-Cancer Analysis of Whole Genomes Consortium, 2020; Robinson et al., 2017; Wu et al., 2022; Zehir et al., 2017).

It is known Ras is implicated in cancer progression and Ras mutations confer significantly poorer prognosis, compared to Ras WT across cancers (Figure 3A). KRAS is most frequently mutated across all cancers (Figure 3B), and there is a vast spectrum of over 200 amino acid substitutions that can occur between all three Ras isoforms, of which G12V and G12D are the most frequent mutations, comprising 45% of all amino acid substitutions in Ras (Figure 3C; Supplementary Table S1). From an isoform perspective, the most frequent substitution in KRAS is G12D, followed by G12V. In contrast, Q61R is the most frequent substitution in NRAS and HRAS, followed by Q61K (Figure 3D). The frequency of isoform mutations is in line with the relative expression of the three isoforms, whereby KRAS is most expressed, followed by NRAS and HRAS (Newlaczyl et al., 2017).

Occurrence and frequency of isoform mutations differ between different types of cancers. Multiple studies converge in finding pancreatic, colorectal and lung cancer most commonly express KRAS mutations (Prior et al., 2012). Over 77% of all pancreatic cancer patients were shown to have KRAS mutations, 42% of colorectal cancer cases have KRAS mutations, and approximately 30% of Non-Small Cell Lung Cancer (NSCLC) cases have KRAS mutations (Hu et al., 2021; Reita et al., 2022; Vaughn et al., 2011; Prior et al., 2012). In contrast, Ras mutations are found in less than 1% of central nervous system cancer cases (Prior et al., 2020). In addition, the incidence of mutations in different Ras isoforms differs between cancers. For example, KRAS mutants dominate the Ras mutational burden in pancreatic cancer and NSCLC, whereas NRAS is most mutated in melanoma and acute myeloid leukaemia. In contrast, HRAS is the most mutated isoform in head and neck cancer (Cerami et al., 2012; Gao et al., 2013; Hyman et al., 2018; ICGC/TCGA Pan-Cancer Analysis of Whole Genomes Consortium, 2020; Miao et al., 2018; Robinson et al., 2017; Wu et al., 2022; Zehir et al., 2017; Prior et al., 2020).

Generally, the most expressed Ras isoform in a cell is the most likely isoform to be mutated, however Ras mutations occur even when Ras expression is considered low in tissues (Hood et al., 2023; The Human Protein Atlas, 2023). For example, KRAS mutations occur in over 90% of pancreatic cancer cases, however is considered to have low expression in the pancreas, compared to other organs (The Human Protein Atlas, 2023; Cox et al., 2014).

The most commonly mutated amino acids across all isoforms, considered hotspot mutations, are G12, G13, and Q61, all occurring within the effector binding region (Figures 1C, 3C). Yet, there is considerable variation in the specific amino acid substitutions at each hotspot (Figure 3D; Supplementary Table S1).

It is crucial to understand the survival risk of different mutations, and whether certain mutations present a greater clinical risk. Although KRAS mutations are most frequent in NSCLC, mutations in other isoforms can impact patient outcome. Clinical data from NSCLC patients participating in the BATTLE trial (carried out in NCT00409968, NCT00411671, NCT00411632, NCT00410059, and NCT00410189) revealed distinct differences in median overall survival (mOS) between patients with different Ras mutations (Kim et al., 2011). KRAS G12C or G12V mutations were shown to confer poorer response to the small molecule kinase inhibitors sorafenib, vandetanib and erlotinib and consequent survival outcomes compared to other KRAS mutations, including KRAS G12D and G12A (Ihle et al., 2012). Progression-free survival (PFS) was approximately half the time in the KRAS G12C and G12V mutated patients, presenting 1.85 months of median survival post-therapy, when compared to 3.35 months in those with other KRAS mutations or wild-type KRAS, where patients exhibited of 1.95 months (Ihle et al., 2012; Kim et al., 2011). Ultimately, this suggests that the G12C mutation plays a particularly key role in the pathogenicity of oncogenic KRAS in NSCLC (Hunter et al., 2015). Such allelic-risk differences are also starting to be examined in other cancers, including colorectal (Zhang et al., 2024).

The clinical impact of the KRAS mutational signature differs in metastatic colorectal cancer, for example (Tejpar et al., 2012). KRAS G13D conferred poorer prognosis than all other KRAS mutants when patients received chemotherapy alone, with a mOS of 14.7, compared to 17.7 in patients with any other KRAS mutation, or 19.7 months in KRAS WT patients. However, once the anti-EGFR antibody cetuximab was added to the treatment regimen, mOS in KRAS G13D patients increased to 15.2 months, and 23.5 months in KRAS WT patients. Contrastingly, mOS decreased by approximately 2 months in patients with other KRAS mutations, once cetuximab was added to the regimen, thus indicating KRAS G13D conferred the most favourable prognosis in metastatic colorectal cancer patients treated with cetuximab and chemotherapy (Tejpar et al., 2012).

## Mutation impact on Ras structure and activation

Mutant Ras can regulate differing cellular events, depending on their context, with it having been previously described that “not all Ras mutations are created equally,” based on the oncogenic properties, likelihood of occurrence and downstream signalling (Miller and Miller, 2011).

Since G12 and G13 are located within the P loop (amino acid 10–17), which is required for coordination of the phosphate groups in the GDP binding site and interaction with GEFs (Zhang et al., 2019b) mutations here can affect Ras structure and downstream signalling, due to structural alterations to the effector binding domain (Figures 1A, B) (Boriack-Sjodin et al., 1998). Similarly, given Q61 is in the Switch II region, mutations here can also cause structural changes (Gebregiworgis et al., 2024; Prior et al., 2020) (Figure 1C). Further to different isoforms having varied selectivity for particular GEFs and GAPs (Bollag and McCormick, 1991; Jones and Jackson, 1998), some Ras mutations can cause GEF-independent activation of Ras. For example, KRAS and HRAS G13D can be activated independently of SOS, due to the negative charge conveyed by the aspartate (Hunter et al., 2015; Chen et al., 2013).

Alterations at G12 and G13 can also impact the conformation of the Switch II region, and can alter the conformation of this region, thereby inhibiting the interaction between the arginine finger (R789) on Ras-GAPs and the Q61 residue, which is essential for stabilising the transition between the GTP-bound and GDP-bound states (Privé et al., 1992; Kötting et al., 2008; Scheffzek et al., 1997). Overall, these structural changes have been shown to affect intrinsic GTPase activity: in KRAS G12A, G12R, Q61H or Q61L, the GTP hydrolysis rate was over 40-fold lower than KRAS WT. In contrast, the KRAS G12C and NRAS G13D mutations have been shown to have little effect on the intrinsic hydrolysis rate, compared to their WT counterparts (Hunter et al., 2015; Smith et al., 2013). Intrinsic nucleotide exchange is instead favoured in some instances, with NRAS G13D and Q61L showing increased intrinsic exchange (Smith et al., 2013).

To facilitate transition from the GTP-bound state to the GDP-bound state, a water molecule causes the hydrolysis of the GTP γ-phosphate by a nucleophilic substitution reaction (Maegley et al., 1996; Pai et al., 1990). This occurs through a structural inversion at the γ-phosphate site, which facilitates the accumulation of a hydrophobic cluster, favouring Ras to be in its transition state and ultimately the non-catalytic (GDP-bound) state (Buhrman et al., 2007).

Furthermore, the oncogenic transforming capability increased over 100-fold in the presence of some Q61 mutants, with HRAS Q61V and Q61L exhibiting the greatest level of activity of 17 different HRAS-Q61 mutants, compared to WT, In contrast, HRAS Q61G had 200-fold lower transforming capability than that of the Q61V mutant (Der et al., 1986). The increased transforming capability of HRAS Q61L was accompanied by a 10-fold decrease in GTP hydrolysis activity compared to HRAS WT. However, the inverse relationship between increased transforming capability and decreased GTPase activity was inconsistent, and instead it appeared that any deviation from the wild-type conferred a decrease in GTPase activity (Der et al., 1986). Further investigation has shown that some Q61 mutants are less amenable to being in their GTP-bound state than others, such as KRAS Q61E and Q61P, which instead may cause their reduced transforming potential despite their increased GTP hydrolysis rate, and in fact KRAS Q61 mutants with less transforming potential have a greater structural similarity to KRAS WT (Huynh et al., 2022; Frech et al., 1994). Indeed, those with a greater transforming potential appear to have a greater aliphatic side chain, which is believed to strengthen the Ras/Raf interaction and lock Ras in its transition state since they contribute to the hydrophobic cluster, therefore reducing GTPase activity (Buhrman et al., 2007).

## Mutant-dependent signalling alterations

The siREN study, investigating Ras-effector interactions in KRAS-mutant cancer, used single cell analysis to interrogate cell viability, reactive oxygen species generation, growth, proliferation and cell death associated with effector knockdown. Ras effectors were considered in “nodes,” whereby knockdown of an effector encompassed all possible isoforms (Yuan et al., 2018). Inhibition of most Ras effector nodes decreased cell viability in more than 80% cell lines tested (KRAS WT or mutant), apart from PDK, Ral effectors, non-canonical NFκB-related Ras effectors, PLCE and PAK (Yuan et al., 2018). There was no differentiation in the effects of node knock-out in KRAS WT or mutant cell lines, indicating the need for treating individual mutants as their own entity, rather than “KRAS-mutant cancer.” Instead, tissue of origin seemed to more closely associate with node sensitivity. Overall, two subgroups of KRAS-mutant cell lines emerged: those depending on KRAS and the other Ras isoforms, and those resistant to KRAS knockout, and which were instead dependent on RSK p90 S6 kinase (RSK) (Yuan et al., 2018). This corresponded to an increased level of signalling through the MAPK pathway in the KRAS dependent cell lines, whereas RSK-dependent cell lines showed increased signalling through components of the PI3K pathway, including AKT and mTOR, but not PI3K itself. Two cell lines did not correlate with KRAS or RSK dependency, and instead exhibited Ral pathway dependence. Enhanced analysis revealed RSK dependency in KRAS-mutant cell lines correlates with *STK11* co-mutation, whilst Ral pathway dependence correlates with *CDKN2A* mutation in KRAS-mutant cell lines, ultimately indicating it is co-mutations which can determine Ras-effector pathway activation (Yuan et al., 2018).

Phospho-proteomic analysis has identified >1,100 proteins that are differentially regulated in NRAS-G12V and Q61L-mutant melanoma (Posch et al., 2016). The G12V mutant melanoma showed a greater level of signalling through the PI3K-AKT pathway by PIM2 phosphorylation, whereas the Q61L mutant showed a greater activity within the MAPK pathway, determined by increased MEK phosphorylation levels (Posch et al., 2016).

*TP53-*knockout bronchial epithelial cell lines were used as an isogenic background to examine effects of KRAS mutations (Ihle et al., 2012). These indicated a preference for Ral pathway activation in a KRAS G12C context, whereas a KRAS G12D context promoted phosphorylation of AKT at Ser473 and Thr308 and mitogen-activated protein/extracellular signal-regulated kinase (MEK) at Thr202 and Tyr 204 (Ihle et al., 2012). Effects of KRAS G12C have elsewhere been associated with Ral activation, with proliferation of KRAS G12C-mutated NSCLC *in vitro* and *in vivo* relying on the Ral pathway, though this is not Ral isoform specific (Yan and Theodorescu, 2018; Yan et al., 2014). Ral-selective allosteric inhibitors BQU57 and RBC8, which are selective for Ral-GDP, have been shown to prevent downstream signalling, colony formation and xenograft-tumour growth of a human lung cancer cell line H2122, which is KRAS G12C and p53-mutated (Yan et al., 2014; Phelps et al., 1996). Increased Ral activity has also been shown in KRAS-4B G12V-mutant pancreatic cancer, due to altered binding kinetics and a more dynamic interaction between KRAS-4B G12V and the RalGEF Rgl2, compared to the KRAS WT interaction with Rgl2 (Tariq et al., 2024). This RalGEF also has potential to be targeted therapeutically, including the targeting of C284 on the RalGEF Rgl2. Covalent binding of indoline-based fragments to this site causes inhibition of Ral GTPase activation, through allosteric inhibition of the Rgl2-Ral interaction (Bum-Erdene et al., 2022).

When considering the reason behind the “favouring” of different Ras-mediated pathways by different Ras isoforms and mutants, it is necessary to consider the interface at which Ras interacts with its effector. KRAS G12V and Q61H must interact with C-Raf exclusively to elicit downstream signalling in the MAPK pathway and overall cellular proliferation (Terrell et al., 2019). Ras localisation is also important in Ras/Raf-mediated signalling, with Bioluminescence Resonance Energy Transfer (BRET) assays showing reduced fluorescence when Raf is overexpressed with KRAS Q61R mutated at the farnesylation site, suggesting that Ras requires correct localization to the membrane to interact with Raf. Taken together, this indicates a propensity of Ras mutations to not only favour the different downstream pathways (e.g., PI3K or MAPK) (Drosten and Barbacid, 2020), but also potential for nuances within these pathways depending on Ras mutational status and show Raf as a potential target to prevent Ras-mutated oncogenic signal.

KRAS-G12 mutations confer different binding affinities for the Ras Binding Domain (RBD) of C-Raf. The KRAS G12D mutant decreased C-Raf-RBD affinity for KRAS approximately five-fold and the G12V mutant decreased Raf affinity by approximately eight-fold compared to KRAS WT, potentially indicating reduced signalling through the MAPK pathway when these mutants are present. In contrast, KRAS G12D tumours, which exhibited a much stronger level of Protein kinase B (AKT) phosphorylation compared to those expressing KRAS WT or Q61H (Zhou et al., 2020). This is not necessarily codon-specific however, since the KRAS G12C did not confer a significant increase or decrease in binding affinity (Zhou et al., 2020).

KRAS-Q61H preferentially binds to C-Raf, due to altered conformation of the Switch II region, which stabilises the switch I region. As seen in Figure 1A, the effector binding region and the nucleotide binding region is located within the switch I region, and therefore stability is essential to permit sufficient Ras activation and Raf binding (Prior et al., 2012; Sharma et al., 2024). Q61H lies within the switch II region, and therefore mutations here can impact the overall structure of Ras and its stability in the GTP or GDP bound state, due to accessibility of the γ phosphate, which is hydrolysed in the transition between these two states (Buhrman et al., 2007; Der et al., 1986). As a result of its preference for C-Raf effector binding, KRAS Q61H NSCLC samples exhibited a greater level of ERK activity, compared to KRAS WT or G12D in the same cohort of NSCLC patient samples (Zhou et al., 2020). This is because of increased flexibility of the Switch II Region in KRAS Q61H, impeding the interaction between the activating p110 PI3K subunits, specifically α and γ, but enhanced interactions with the Ras Binding Domain (RBD) on Raf (Zhou et al., 2020). Similarly, HRAS Q61L has also shown increased flexibility of the SII region compared to WT, which stabilises the Ras-Raf interaction, and the downstream Raf-MEK interaction (Hobbs et al., 2016; Buhrman et al., 2007; Fetics et al., 2015).

Alterations in nucleotide binding capabilities has also shown to be responsible for the increased tumorigenesis in melanoma. *Ex-vivo* comparison of the NRas Q61R and NRas G12D mutants in allelic knock-in mice revealed NRas Q61R was significantly more tumour-promoting and showed a significantly lower in rate of nucleotide exchange, with a stronger propensity to remain in its GTP-bound state (Burd et al., 2014). This was initially believed to be the reason for the increased melanomagenic properties seen in Q61-mutant mice, rather than increased effector binding, since B-Raf binding affinity was only four-fold greater in NRas Q61R mice, compared to NRas WT mice, and there did not appear to be a significant codon-specific change in MAPK/ERK pathway activation (Burd et al., 2014). NanoBiT and BRET assays subsequently revealed NRas Q61R and Q61K had a greater affinity for B-Raf compared to WT, which may explain the increase in MAPK signalling in such circumstances–however this effect is not purely driven by the codon which is mutated, as NRas Q61P did not increase MAPK/ERK signalling to the same degree, or exhibit decreased GTPase activity (Murphy et al., 2022). PI3K binding affinity did not significantly increase in binding affinity in KRAS G12D, NRAS G12D or NRAS Q61R-mutant *in vitro*, however there was a significant decrease in AKT phosphorylation at Ser473 in NRAS Q61-mutant melanoma cell lines, compared to NRAS G12 or G13 mutant cell lines, which was used as a marker for PI3K pathway activation (Burd et al., 2014). This may suggest activation of the MAPK pathway could supersede the need for PI3K engagement in these cell lines, although thus far remains undetermined.

Further to this, increased susceptibility to pharmacological MAPK/ERK kinase 1/2 (MEK1/2) inhibitors selumetinib, trametinib and RAF709 was seen in KRas-Q61H mice, compared to KRas-G12D (Zhou et al., 2020). Comparable results were seen in other KRAS-Q61H lung cancer cell lines in a separate study (Choi et al., 2019). It has since been indicated that concomitant inhibition of SOS1 can improve sensitivity to MEK inhibition in a G12 and G13 mutant KRAS context, but this is not seen in KRAS-Q61 mutant cases of lung cancer. However, knockout of KSR1 synergises equally between mutants to enhance activity of MEK inhibitors (trametinib), which has been suggested to be a result of KSR1 acting independently of any GEF-mediated activity (Hunter et al., 2015; Daley et al., 2023b). Q61 mutants are not reliant on GEF activity for their oncogenic properties, instead conferring a greater Ras activity through a lack of intrinsic hydrolysis (Hunter et al., 2015).

Sensitivity of NRAS Q61-mutant tumours to MEK inhibitors has also been shown clinically. During the NCI-MATCH trial (NCT02465060) investigating the use of the MEK inhibitor Binimetinib, a more favourable response was seen in NRAS-Q61-mutant colorectal cancer patients compared to those with NRAS-G12 or G13 mutations, with a median overall survival (mOS) of 15 months versus 5.1 (Cleary et al., 2021). This also correlated with an increased sensitivity of NRAS-Q61-mutant cell lines to other MEK inhibitors such as trametinib and selumetinib, which was determined using the Genomics of Drug Sensitivity in Cancer database (Cleary et al., 2021).

## Effects of mutations in Ras-effectors

The occurrence of mutations in Ras effectors in conjunction with mutated Ras also impacts downstream effector signalling. Class I and Class II mutations in BRAF typically occur exclusively from KRAS, and they are considered constitutive signalling activators. Class I mutants, such as the most common V600E mutation, confer increased kinase activity in their monomeric form, whereas Class II mutants, such as K601E, require homodimer formation, to promote increased kinase activity (Davies et al., 2002; De Roock et al., 2010; Yaeger and Corcoran, 2019; Sforza et al., 2022). Class III mutations, such as D287H however, are considered amplifiers, needing to occur with upstream mutations, such as Ras isoform mutations, eliciting their effect by amplifying upstream activation, which is often caused by KRAS mutations. Class III mutations themselves have impaired kinase activity (Yaeger and Corcoran, 2019). As such, KRAS and B-RAF mutations are largely considered to be mutually exclusive, due to the overwhelming instance of B-RAF Class I mutations (85% in the GENIE v13.1 Public Cohort), compared to Class II or III (De Roock et al., 2010; Liu and Xie, 2023; Garcia-Carbonero et al., 2020).

B-RAF mutant cancer cell lines have been shown, as expected, to be highly responsive to MEK inhibition but not AKT inhibition, whereas KRAS-mutant cell lines show almost equal sensitivity to both MEK and AKT inhibition. However, some KRAS-mutant cell lines were not sensitive to either, indicating underlying mechanisms outside of the canonical MAPK and ERK pathways (Stewart et al., 2015). Whilst activation of both KRAS and B-RAF can stimulate downstream ERK activation, its aberrant activation is not necessarily solely reliant on B-RAF or KRAS mutations, since ERK activity was seen to be not significantly different between WT or mutated B-RAF or KRAS in melanoma, and other ERK regulatory mechanisms are thought to be involved (Houben et al., 2008).

Mutational exclusivity between KRAS and B-RAF mutations can cause variations in the immune response to tumours, with KRAS-mutant tumours exhibiting a lower rate of immune cell infiltration compared to WT, whereas B-RAF mutations caused an increase in immune cell infiltration, compared to WT (Edin et al., 2024). This was accompanied by a positive prognostic effect in colorectal cancer, with increased immune cell infiltration conferring a better prognosis (Edin et al., 2024).

Whilst it is known that PI3K is essential in mediating the effects of oncogenic Ras and supporting tumour maintenance (Castellano and Downward, 2011; Lim and Counter, 2005), the incidences of co-mutations between PI3K pathway and Ras differs between cancers. 60% of endometrial cancer cases are co-mutated in KRAS and *PI3KCA*, yet only 7% of colorectal cancer patients exhibit these co-mutations (Oda et al., 2008; Voutsadakis, 2023). Mutation hotspots E542K, E545K and H1047R in the *PI3KCA* gene account for approximately 80% of all mutations within this gene and are considered gain-of-function mutations, typically occurring when tumours are in their invasive state (Castellano and Downward, 2011; Oda et al., 2008). While the RalGEF, MAPK, and PI3K pathways are all necessary to promote tumorigenesis, activation of the PI3K pathway has been shown to promote tumour maintenance to a greater extent than the other Ras effector pathways, particularly by AKT activation by the PI3K p110α isoform (Lim and Counter, 2005). Activating mutations in PI3K has been shown to enhance tumorigenicity in *in vitro* endothelial cell models and colorectal cancer cell lines, compared to KRAS mutations alone (Oda et al., 2008).

However, PI3K pathway mutations in conjunction with KRAS G12C mutations in patients do not confer poorer survival outcomes in response to KRAS G12C inhibition in a large cohort of KRAS G12C-mutated NSCLC patients. Although progression-free survival significantly decreased in patients with PI3K pathway mutations, this did not confer a significantly poorer overall survival (Negrao et al., 2023a). In contrast, other (non-KRAS G12C) Ras mutations did confer significantly poorer progression-free and overall survival, by 2 months and 7 months, respectively (Negrao et al., 2023a). It must be noted, however, that this study did not publish data on the individual mutations, only a cluster of genes within a pathway.

It has previously been reported that wild-type PI3K, and more specifically the p110α subunit, is essential in mediating KRas-G12D-driven lung cancer formation in mouse models, with mutations in the Ras Binding Domain of PI3Kα inhibiting oncogenesis (Gupta et al., 2007; Engelman et al., 2008). These studies showed a synergy between inhibiting the MAPK and PI3K pathways in KRas-G12D-driven carcinogenesis, with increased tumour eradication in mice treated with inhibitors of both pathways, including the PI3K/mTOR dual inhibitor NVP-BEZ235 and the MEK inhibitor ARRY-142886 (Engelman et al., 2008; Gupta et al., 2007). In this example, EGF or PDGF-stimulation exhibited differing likelihoods for downstream pathway activation, which also depended on PI3K mutational status. Homozygous T208D and K227A mutations within the p110-α Ras-binding domain resulted in decreased AKT phosphorylation but did not change ERK phosphorylation, indicating reliance on this PI3K isoform to stimulate the downstream signalling, as expected (Hamad et al., 2002; Gupta et al., 2007). This signalling appears to control certain developmental processes in KRas-G12D mice, with lymphatic development attenuated, and cell cycling was reduced (Gupta et al., 2007). Moreover, the oncogenic properties of KRas-G12D were found to rely on the KRAS-PI3Kα interaction, since oncogenesis was significantly inhibited, with tumour cells undergoing apoptosis before the formation of macroscopic tumours (Gupta et al., 2007). This indicates KRas-G12D lung adenocarcinoma in the KRAS LA2 model used here relies on PI3K signalling.

Altogether, the picture of Ras mutations in cancer and other diseases is highly variable and therefore its role in disease is justifiably important. Ultimately though, the reasons for these different amino acid alterations at these various positions, alongside their differing phenotypic effects, remains to be fully elucidated.

## The Ras therapeutic revolution – Targeting cysteine

**FIGURE 4:**
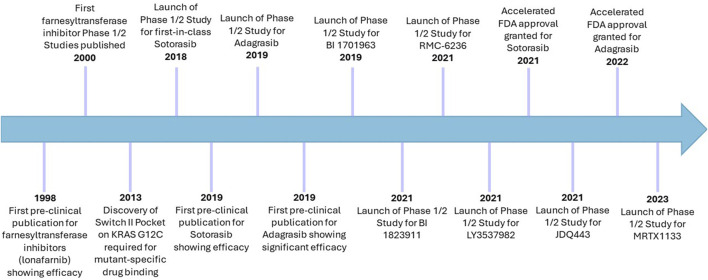
Timeline of cysteine-targeting direct Ras inhibitors in cancer. Sotorasib and adagrasib remain the only two FDA-approved direct Ras targeting therapies, which are both G12C-mutant specific. Despite initial challenges with farnesyltransferase inhibitors, these remain in clinical trials for cancer, albeit more selectively than before. Lonafarnib has already been approved for use in Hutchinson-Gilford progeria syndrome (2021).

Ras has previously been considered undruggable, however was revolutionised by the development and FDA-approval of KRAS-G12C-mutant inhibitors (Figure 4) (Marensi, 2024; Ostrem et al., 2024). Deeper understanding of a previously considered non-targetable groove around the Switch II region using *in silico* and *in vitro* work determined this pocket was pharmacologically targetable and was termed SII-P (Ostrem et al., 2013; Milburn et al., 1990). Using large-scale fragment screening targeting the covalent nature of the G12C mutation which occurs in SII-P, compound 6H05 was identified as the first hit for a direct KRAS G12C inhibitor (Ostrem et al., 2013). This was subsequently pharmacologically optimised, generating compounds ARS853 and ARS1620. These pre-clinical compounds lock KRAS-G12C in an inactive GDP-bound state through occupation of the γ phosphate binding location, preventing phosphorylation of MEK, ERK, RSK and AKT, and ultimately arresting cellular proliferation (Janes et al., 2018; Patricelli et al., 2016; Lito et al., 2016). Subsequent optimisation following *in vivo* work led to development of AMG510 (sotorasib), which eventually became the first-in-class FDA-approved KRAS G12C inhibitor, less than a decade after the Shokat lab drug binding site breakthrough (Punekar et al., 2022; Janes et al., 2018; Skoulidis et al., 2021) (Figure 4). Other compounds have since been developed to target the SII-P binding pocket, as well as other Ras interfaces, including the second FDA-approved KRAS G12C inhibitor MRTX849 (adagrasib) (Hallin et al., 2020). Supplementary Table S2 indicates the clinical trials in which these compounds have been tested and presents the current state of direct Ras inhibitors in trials.

Sotorasib was the first approved by the FDA as a second-line treatment for adults with locally advanced or metastatic non-small cell lung cancer (NSCLC) with confirmed KRAS G12C mutations, following the success of the CodeBREAK100 trial, as well as many other CodeBREAK trials (NCT03600883) (Liu et al., 2023). This Phase I clinical trial showed a disease control rate of 88.1%, a median progression-free survival (mPFS) of 6.3 months and confirmed the optimal dose of 960 mg once daily in dose-escalation studies (Hong et al., 2020). Improved success was seen in the subsequent phase II trial, where there was an mOS of 12.5 months, a disease control rate of 80.6% and a mPFS of 6.8 months (Skoulidis et al., 2021). Most common treatment-related adverse effects were fatigue, diarrhoea and nausea, which occurred in ∼70% patients. Despite that, the overall success of this phase II trial in conjunction with the necessity for this new class of therapeutics resulted in accelerated FDA approval in 2021 (Skoulidis et al., 2021).

A subsequent trial has compared the use of sotorasib to standard of care chemotherapy, assessing potential benefits from the use of targeted therapy. The Phase III trial NCT04303780 compares sotorasib to docetaxel, in patients with previously-treated advanced metastatic NSCLC harbouring G12C mutation (de Langen et al., 2023). Results to date indicate a significantly increased mPFS from 4.5 months, with docetaxel, to 5.6 months in the sotorasib-treated group. Sotorasib is better tolerated in patients, with reduced incidence of grade 3+ treatment-related adverse effects (40% with docetaxel, 33% with sotorasib) (de Langen et al., 2023).

Adagrasib (MRTX849) is a KRAS G12C inhibitor with improved pharmacokinetics, shown to cause less hepatotoxicity than sotorasib (Luo et al., 2024). Adagrasib was also approved in 2021 as second-line monotherapy for adults with KRAS-G12C mutated NSCLC (Nakajima et al., 2022) The KRYSTAL-1 Phase I trial (NCT03785249) was a dose escalation study where the patients with any solid tumour, harbouring a KRAS-G12C mutation received oral treatment and presented a mPFS of 6.5 months (Ou et al., 2022). Phase II KRYSTAL-2 trials (NCT04330664) further combined adagrasib with TNO155, a selective inhibitor of Src homology-2 domain-containing protein tyrosine phosphatase-2 (SHP2) to treat patients with advanced solid tumours that have a KRAS-G12C mutation to prevent drug resistance through ERK1/2 signalling (Sabari et al., 2021). Given the benefits seen thus far, the KRYSTAL trial repertoire has been considerably expanded, including investigations of the use of adagrasib in combination therapy. This includes combination with anti-PD1 pembrolizumab in advanced/metastatic solid tumours with KRAS-G12C mutations (NCT04185883 and NCT03785249), as well as other MAPK pathway inhibitors, such as ERAS-007 (NCT04959981).

However, all trials thus far are in solid tumours. Whilst research suggests that it can be highly beneficial to other cancers, such as acute myeloid leukaemia, strongly driven by Ras mutations. Therefore there remains an unmet clinical need to expand these trials even further (Healy et al., 2022).

In comparison to the vast advancement being made to target KRAS pharmacologically, the direct targeting of other KRAS mutations, as well as HRAS and NRAS mutations is significantly behind, mostly due to the lack of an available cysteine. As result, there are no drugs approved or in clinical trials for direct NRAS or HRAS targeting, leading to a distinct necessity to identify new drug targeting sites. Nevertheless, the flexibility of certain mutation-specific inhibitors to act on multiple Ras isoforms could lead to therapeutic advances. Although sotorasib was considered KRAS G12C specific and is FDA-approved for KRAS G12C mediated cancers only (Skoulidis et al., 2021), studies have indicated it may be suitable for other NRAS or HRAS G12C-mutated cancer, delivering potential to revolutionise the way in which the drug can be used. In an isogenic Ba/F3 cell line engineered to over-express G12C mutations in KRAS, NRAS or HRAS, the IC_50_ for sotorasib was five-fold lower in the NRAS G12C model than that for KRAS G12C or HRAS G12C model. IC_50_ values for KRAS G12C and HRAS G12C were comparable (Rubinson et al., 2024). Such increased potency was attributed to the presence of L95 in NRAS, compared to H95 in KRAS or HRAS, since generation of the L95H mutation in the NRAS G12C isogenic cell line reduced sotorasib sensitivity five-fold (Rubinson et al., 2024; Mahran et al., 2024; Koga et al., 2021; Fell et al., 2020). In contrast, interaction at H95 is essential for the binding and efficacy of other KRAS G12C inhibitors, such as adagrasib, explaining its inefficacy in NRAS-G12C mutant contexts (Rubinson et al., 2024). Sotorasib has also shown clinical efficacy in an NRAS-G12C colorectal cancer patient, whose tumour burden decreased following sotorasib therapy, as determined by serum markers and CT imaging. This was accompanied by a decrease in percentage NRAS G12C mutant alleles, which were eliminated after 80 days of therapy (Rubinson et al., 2024), thereby indicating potential to widen the clinical use of this drug.

Other KRAS-G12C inhibitors are being developed and undergoing preclinical or first-in-human trials, aiming to improve upon the potency and efficacy of current options. LY3537982 has over 10-fold greater potency compared to sotorasib and adagrasib, when considering KRAS GTP loading inhibition, and tumour regression (Peng et al., 2021). Indeed, promising *in vivo* mouse tumour regressions were seen and as such, this compound has now proceeded to Phase I/II trials as a monotherapy (NCT04956640) or in combination with immunotherapy and/or chemotherapy (NCT06119581) (Peng et al., 2021; Kwan et al., 2022).

JDQ443 is a KRAS G12C inhibitor, which also locks KRAS G12C in its inactive, GDP-bound state, in a similar mechanism to other KRAS G12C inhibitors (Weiss et al., 2022). However, its structure and binding within SII-P differs from sotorasib and adagrasib. and it does not interact with the H95 residue on KRAS, leading to potential for other Ras G12C-mutant cancers (Weiss et al., 2022). This compound inhibits ERK activation tumour growth in KRAS G12C pancreatic cell line derived xenograft mice. The difference in tumour size after 16 days was ∼12-fold, in JDQ443-treated mice, compared to vehicle-treated. In contrast, KRAS G12V pancreatic cancer cell line derived xenograft mice did not show any tumour inhibition over this period (Weiss et al., 2022). Following this successful preliminary data, clinical trials have started to examine JDQ443 in solid cancers, including the KontRASt-02 study comparing the use of JDQ443 with docetaxel in NSCLC (NCT04699188, NCT05358249, NCT05132075).

BI-0474 is a KRAS G12C inhibitor, binding in SII-P, but with structural capability to act in a non-covalent manner (Bröker et al., 2022). The addition of a covalent warhead permitted this to act at G12C, eliciting anti-proliferative effects in the NCI-H358 KRAS G12C mutant lung cancer cell line that were comparable to that of sotorasib (26 nM potency) (Bröker et al., 2022). Indeed, this compound successfully inhibited tumour growth in cell line derived xenograft mice, inhibiting ERK phosphorylation and inducing apoptosis (Bröker et al., 2022).

BI-1823911 has also recently entered phase I clinical trials (NCT04973163) (Savarese et al., 2021). It proved to exert an effective anti-proliferative response in preclinical studies across a range of *in vitro* and *in vivo* models, with a similar or increased potency in comparison to currently approved KRAS-G12C inhibitors sotorasib and adagrasib. BI-1823911 initiated a sustained inactivation of MAPK pathway signalling, measured using ERK activation as a signalling pharmacodynamic biomarker, which correlated with downregulation of MAPK-pathway responsive genes, including *CCND1* and *DUSP6* (Savarese et al., 2021). This correlates with the perceived favouring of the MAPK pathway by the G12C mutant, as described earlier in this review (Kim et al., 2020). Further pre-clinical studies suggested that efficacy could be further increased when administered as a combination therapy with BI-1701963, a KRAS-SOS1 interaction inhibitor. Resultantly, BI-1823911 is now in Phase I clinical trials as a monotherapy and in combination with BI-1701963, a KRAS-SOS1 inhibitor, to investigate its use against advanced/metastatic solid tumours (Savarese et al., 2021).

GDC-6036 (divarasib) is another covalent G12C inhibitor, developed by Genentech (Meng et al., 2022). This, like sotorasib, adagrasib and JDQ443, acts to lock KRAS G12C in its inactive state (Meng et al., 2022; Weiss et al., 2022; Janes et al., 2018). This has shown high anti-tumour potency in KRAS G12C pancreatic cancer cell line derived mouse models, with mass spectrometry analysis revealing significant decreases in MAPK pathway activation (Tran et al., 2023). This correlates with a decrease in circulating tumour DNA detected within NSCLC and CRC patients, which itself was seen to correlate with response to divarasib (Choi et al., 2024). 53% of NSCLC patients exhibited a confirmed response in a Phase I/II trial (NCT04449874), whilst this was 29% for CRC patients (Sacher et al., 2023). Median PFS was 13.1 months for NSCLC patients, and 5.6 months for CRC patients (Sacher et al., 2023).

RMC-6291, another covalent G12C inhibitor, acts through formation of a complex with KRAS G12C and cyclophilin A, which inhibits binding of Ras effector proteins to KRAS G12C (Jänne et al., 2023). This has been reported to show more durable responses than other KRAS G12C inhibitors such as adagrasib (Cregg et al., 2023). Early data from Phase I trials indicate a 44% overall response rate in patients who had never received directed KRAS G12C therapy, increasing to 57% in those who had recently received KRAS G12C therapy (Jänne et al., 2023). Toxicity was able to be ameliorated with dose reduction (Jänne et al., 2023).

Aside from G12C mutant targeting, cysteine necessary for Ras post-translational modifications has also been examined.

Preventing Ras farnesylation would prevent it from trafficking to the plasma membrane and interaction with receptors and their scaffold proteins to engage in signalling, suggesting that targeting the CAAX domain in Ras could be an attractive way to target the oncogenic action of Ras. Moreover, preventing Ras farnesylation could be a strategy to target Ras despite the absence of a G12C mutation.

*In vitro* studies show that inhibition of Ras farnesylation interferes with its activity (Long et al., 2001). Farnesyltransferase (FTase) inhibitors have been in development for cancer therapy for approximately 30 years (Gibbs et al., 1994). Peptidomimetics were developed to prevent binding of the FTase to the CAAX farnesylation site of Ras. The benzodiazepine peptidomimetic CVFM was show to successfully to inhibit the FTase activity and H-Ras function *in vitro*, fibroblasts and oocytes, showing to be specific to farnesylation not geranylgeranylation (Garcia et al., 1993; James et al., 1993; Marsters et al., 1994). However, there are some limitations with peptidomimetics, including poor cellular uptake and degradation by proteases (Wang et al., 2022a).

Tipifarnib is a small molecule effective in preventing Ras prenylation by FTase (Cox et al., 2014). Its efficacy has been shown in HRAS-mutant rhabdomyosarcoma, inhibiting membrane localisation and downstream ERK signalling in cell lines, irrespective of Ras mutation status (Odeniyide et al., 2022). However, phenotypic effects were significantly more pronounced in HRAS-mutant cells than NRAS mutant or Ras wild-type cell lines and cell line derived xenograft models, with overall cytostatic effects seen (Odeniyide et al., 2022). Addition of tipifarnib to existing MAPK inhibitor treatment regimens has shown promise in HRAS-driven thyroid cancer, further inhibiting ERK activation and abrogating HRAS mutant signalling (Untch et al., 2018). Furthermore, tipifarnib confers a strong inhibitory effect in HRAS-driven head and neck cancer, *in vitro*, *in vivo* and latterly in clinical trials. Cell proliferation and colony forming capability of the HRAS G12C ORL-214 head and neck cancer cell line significantly decreased in the presence of 1 nM tipifarnib, but not in HRAS-WT cell lines (Gilardi et al., 2020). HRAS-mutant cell line derived xenograft models also exhibited tumour regression, irrespective of the mutation introduced, however WT mice did not, indicating its effects are restricted to oncogenic HRAS, potentially through reduction in angiogenesis and cell cycling (Gilardi et al., 2020).

In 2023, the FDA conferred tipifarnib the designation of breakthrough therapy, as result of the phase I/II trial (NCT02383927) in patients with HRAS-mutant head and neck squamous cell carcinoma (Ho et al., 2021). Overall survival increased by 1.8 months in patients receiving tipifarnib, compared to the current standard of care. Furthermore, tipifarnib has also been shown to be beneficial in oncogene-addicted cancers (such as NSCLC and metastatic melanoma) when administrated in conjunction with other kinase inhibitors, such as alpelisib, osimertinib, dabrafenib and sunitinib (Delahaye et al., 2022; Figarol et al., 2022; Greenberg et al., 2022; Ho et al., 2021; Smith et al., 2023). There was also a degree of synergy seen between tipifarnib and sotorasib (Delahaye et al., 2022). This could be through inhibition of mToR, whose co-inhibition alongside tipifarnib therapy has been shown to improve anti-tumoral effects and is being pursued in clinical trials (NCT04997902) (Smith et al., 2023). Additionally, lonafarnib was the first farnesylation inhibitor approved by the FDA for clinical use, entering the market in 2020 to inhibit progerin farnesylation in Hutchinson-Gilford, disease (Mullard, 2021).

Targeting the post-translational S-palmitoyl modification of NRAS preventing its membrane localisation and consequently depleting NRAS downstream signalling using C8 alkyl Cysteine has been successful in pre-clinical models (Vora et al., 2020). This reduced cell proliferation in melanoma cell lines and inhibited tumour progression in xenograft melanoma NRas-mutant mouse models (determined through *ex vivo* K_i_67 staining), which was associated with a decrease of ERK phosphorylation (Vora et al., 2020). Although promising results have been presented with limited “off-target toxicity” such as drug-induced liver injury (Vora et al., 2020), targeting Cysteine residues lacks specificity and may interfere with many key proteins, leading to severe side effects by “on-target” toxicity. For instance, many proteases rely on a catalytic Cysteine, suggesting that these would be among the proteins affected by the effect of palmitoylation inhibitors.

## Non-cysteine Ras-targeted therapies in development

Despite the fact KRAS G12C mutations only account for 12% of all KRAS mutations (Figure 3D), this remains the only genotype for which a mutant-specific drug is clinically approved. Adagrasib and sotorasib act through covalent interactions at G12C, thereby disregarding other mutations incapable of forming such covalent interactions (Lanman et al., 2020). Therefore, additional consideration of mutant-specific targeting is essential in addressing the unmet clinical need posed by Ras mutations.

KRAS G12D occurs in 30% of KRAS mutant cancers, making it more common than KRAS G12C mutations and yet there are still no approved pharmacological agents to directly target this (Figure 3D). The Shokat group, in collaboration with Mirati Therapeutics have developed a potent, selective, non-covalent KRAS-G12D inhibitor (MRTX1133) (Zheng et al., 2024). MRTX1133 demonstrated *in vivo* efficacy in tumour models harbouring KRAS-G12D mutations, with greatest preclinical efficacy seen in pancreatic ductal adenocarcinoma (PDAC). This is a high-impact development towards direct Ras targeting, given that KRAS mutations occur in ∼93% of PDAC cases, with ∼42% of those being KRAS-G12D mutated (Li et al., 2018). Elsewhere, MRTX1133 has shown efficacy in numerous independent studies, showing significant inhibition on the MAPK pathway in KRAS G12D mutated cell lines, but conferred no effect on the AKT pathway in these cell lines, and had a lower potency in KRAS WT or KRAS G12C cell lines (Kemp et al., 2023). In PDAC cell line derived xenograft mice, 94% growth inhibition and 73% tumour regressions was seen following 30 mg/kg MRTX1133 treatment, with complete or near-complete remissions in mouse models 14 days post-treatment also described (Wang et al., 2022b; Kemp et al., 2023). Ultimately, successful efficacy and safety pre-clinical profiles of MRTX1133 resulted in initiation of the first in human phase I/II clinical trials in patients with solid tumours harbouring KRAS G12D mutations (NCT05737706).

A second KRAS G12D inhibitor, RMC-9805, is now in clinical trials (NCT06040541). This acts on the GTP-bound form of KRAS G12D, and has induced measurable responses in pre-clinical cell line derived mouse models of KRAS G12D mutated NSCLC and PDAC, although less so in colorectal cancer models (Jiang et al., 2023). Furthermore, the effects of RMC-9805 were enhanced by addition of immunotherapy, or small molecule Ras pathway inhibitors, such as mToR (Jiang et al., 2023).

Pan-Ras inhibitors have previously been designed to target KRAS, NRAS and HRAS simultaneously. DCAI, for example, was an early small molecule pan-Ras inhibitor which bound Ras between the α2 helix and β-sheets (β1- β3), adjacent to the Ras-SOS interaction surface (Maurer et al., 2012). This inhibited the interaction between Ras proteins and SOS-1, thereby inhibiting SOS-mediated nucleotide exchange from Ras and locking Ras in its inactive GDP-bound state, decreasing the phosphorylation of downstream signalling kinases, including ERK and AKT (Maurer et al., 2012).

KYA1797K is a compound which destabilises β-catenin, through activation of the Axin-GSK3β complex (Park et al., 2019). However, this drug also facilitates inhibition of Ras in a non-isoform specific manner, with decreased Ras expression, MEK and ERK phosphorylation seen in acute myeloid leukaemia cell lines following treatment with the compound (Hahn et al., 2019). This compound was also able to overcome EGFR inhibitor resistance in NSCLC, through inhibition of the ERK phosphorylation (Park et al., 2019). Moreover, KYA1797K is also able to suppress AKT phosphorylation, as witnessed in colorectal cancer cell lines, organoid and mouse models (Cho et al., 2020).

RMC-7977 and RMC-6236 employ a different binding strategy to the KRAS G12-mutant inhibitors previously discussed, instead binding to active KRAS, and inhibits Ras-effector interactions (Jiang et al., 2024; Holderfield et al., 2024). Although RMC-7977 does have the ability to form covalent interactions with G12C, it can also form extensive non-covalent interactions, to elicit its inhibitory effects on other KRAS mutants (Holderfield et al., 2024). In response to treatment with RMC-7977, ERK activity across a wide variety of KRAS G12-mutant cells was inhibited, however this was not fully suppressed in cell lines expressing KRAS WT, Q61H, G12A, Q61R, G13D or A146T. Suppression of the Ral and AKT pathways was less consistent (Holderfield et al., 2024). RMC-6236 also exerts activity across a range of KRAS G12 mutations, as well as NRAS Q61 mutations, both *in vitro* and *in vivo*. In G12C, G12D and G12V cancer cell line derived xenograft mouse models, both compounds resulted in tumour regression. Indeed, sensitivity to RMC-6236 was sustained *in vitro* and *in vivo* even in the presence of common secondary mutations which confer resistance to KRAS G12C inhibitors, either within Ras, such as Switch II pocket mutations or oncogenic Ras hotspot mutations, or due to amplification of receptor tyrosine kinases, such as EGFR or HER2 (Holderfield et al., 2024; Jiang et al., 2024; Awad et al., 2021; Tanaka et al., 2021). RMC-6236 has also shown efficacy in patients as a monotherapy, during clinical trials (NCT05379985), where at least two patients, one with metastatic KRAS G12D mutated PDAC and one with KRAS G12V NSCLC exhibited five or more months disease free (Jiang et al., 2024).

An alternative pan-Ras inhibitor, ADT-007 has also shown promise, binding KRAS during its nucleotide-free state, i.e., during the exchange of GDP for GTP. This acts on both KRAS WT, when it is hyper-expressed, and mutant KRAS, including G12D and G12V. It is capable of inhibiting MAPK and AKT pathway activation, leading to inhibition of colony formation *in vitro*, cell cycle arrest and apoptosis in KRAS-mutant or over-expressed cell lines (Foote et al., 2024). This is coupled with inhibition of tumour growth in pancreatic and colorectal cancer mouse models, through modulation of the tumour microenvironment (Foote et al., 2024). Optimisation of ADT-007 led to improved pharmacokinetic properties, generating pro-drug ADT-1004, of inhibiting both MAPK and AKT pathway activation in KRAS G12C, G12D, G12V, and G13Q, and reducing tumour volume in patient or cell line derived xenograft KRas-mutant pancreatic cancer mouse models (Piazza et al., 2024).

Such pathway inhibition promiscuity could lead to considerable drug efficacy, though there are concerns. As detailed previously in this review, multi-pathway inhibition may not be necessary in all cases since certain mutations favour certain kinase pathway activation in certain disease contexts. Pan-Ras inhibition also has the potential to deplete wild-type Ras signalling in non-cancer cells, resulting in toxicity concerns, reiterating the need to optimise development of mutant selective Ras inhibitors (Moore et al., 2020). Compounds such as the aforementioned RMC-7977 appear to show selectivity for mutant or over-expressed, therefore reducing the risk of “on-target” toxicity concerns (Wasko et al., 2024).

In order to target multiple Ras mutations, rather than just the G12C, an inhibitor must not rely on covalent binding. BI-0474 introduced the concept of non-covalent binding within SII-P, and this was utilised by removing the covalent warhead and generating the BI-2865 compound (Kim et al., 2023; Bröker et al., 2022). This exhibited comparable activity to BI-0474, and sotorasib with regards to proliferative signalling (Kim et al., 2023). BI-2865 acts on GDP-bound KRAS, capable of binding a host of KRAS mutations, though had little potency against NRAS or HRAS mutations (Kim et al., 2023). This is in part due to the need to bind H95 which is absent in NRAS, a mechanism like adagrasib, as well as variation in amino acids at positions 121 and 122. In a similar way to sotorasib, BI-2865 binds KRAS in its GDP-bound state, in a similar region to the KRAS G12C inhibitors, though does not extend through the P loop around G12, therefore permitting its promiscuous efficacy across KRAS G12C, G12D, G12V, and G13D (Kim et al., 2023). There was little inhibition of effector binding to KRAS in the presence of the drug, therefore the overall mechanism of action is believed to be through the maintenance of KRAS in its inactive state, rather than effector blockade (Kim et al., 2023). BI-2865 successfully inhibited MAPK signalling in a host of cell lines, but not those containing KRAS G12R, Q61L, Q61K or Q61R mutations, and phenotypic effects included growth inhibition in a subset of KRAS-mutant cell lines, as well as caspase activation (Kim et al., 2023). When this drug was optimised for *in vivo* administration (BI-2493), tumour growth in colorectal cancer or PDAC cell line derived xenograft mouse models carrying KRAS G12C, G12D, G12V or A146V was slowed, and a tolerable toxicity profile seen (Kim et al., 2023).

*In silico* design has presented many opportunities for the development of non-G12C, mutant-specific compounds, including those targeting G12S, G12R and Q61R (Hu and Martí, 2024; Zhang et al., 2022a; Zhang et al., 2022b). The Shokat group have identified means of manipulating binding within SII-P to selectively target G12R, targeting the weak nucleophilicity of the arginine with α,β-diketoamides, forming covalent interactions (Zhang et al., 2022b). However, this compound is only capable of binding inactive KRAS G12R, and therefore must be optimised for the active form, given its high frequency in this state (Zhang et al., 2022b). In contrast, G12S has been targeted using acylation of the serine, using β-lactone compounds (Zhang et al., 2022a). These were shown to suppress both ERK and AKT phosphorylation in KRAS G12S homozygous and heterozygous cell lines, whilst exhibiting little effect on KRAS WT cell lines (Zhang et al., 2022a). Although arginine and serine are less conventional covalent-binding targets, their weak nucleophilicity has, in these instances, been utilised in therapeutic targeting, presenting opportunity for further mutant-specific inhibition.

Targeting of NRAS-Q61 remains in its infancy, although isomer-sourced structure iteration (ISSI) *in silico* design has recently suggested compounds capable of NRAS-Q61-mutant binding. A potent, selective inhibitor of NRAS-Q61R, HM-387, is the first of its kind, and utilises hydrogen bonding to simulate the inactive form of NRAS-Q61R (Hu and Martí, 2024). Although this is yet to reach an *in vitro* testing stage, identification of such compound, which may also have an effect on other positively-charged mutations, presents a further avenue of future investigation (Hu and Martí, 2024).

## Emergence of resistance to targeted Ras therapies

Although Ras targeting agents are still new to the market, resistance is starting to be seen. Adagrasib resistance is already a clinical problem, yet exact mechanisms by which resistance develops are not yet fully elucidated. In the KRYSTAL-1 adagrasib monotherapy trial (NCT03785249), of all the patients who exhibited sustained disease progression determined to be due to acquired adagrasib resistance, putative mechanisms of resistance could only be determined in 45% (Awad et al., 2021). Elsewhere, KRAS G12C inhibitor resistance was deemed to be highly heterogenous even in cell line based models of KRAS inhibitor resistance, suggesting the need for a multi-faceted approach (Xue et al., 2020). This included, in some cases, the acquisition of secondary mutations post treatment. Specifically, KRAS G12D/R/V/W, G13D, Q61H, R68S, H95D/Q/R, Y96C, and Y96D secondary mutations were identified to be associated with resistance in these patients, potentially due to clonal expansion of minor subclones (Awad et al., 2021; Zhao et al., 2021). Y96D is believed to disturb the binding between Y96 which occurs in the SII-P and the inhibitor and has been suggested to confer resistance to adagrasib as well as sotorasib and its derivative ARS-1620 (Tanaka et al., 2021). However, such resistance could potentially be overcome using the novel inhibitor RM-018, which again is specific for KRAS G12C. However, it acts at a different site to adagrasib and sotorasib, instead binding KRAS G12C in its GTP-bound active state, forming a complex with cyclophilin A, in a similar manner to RMC-6291 (Tanaka et al., 2021; Jänne et al., 2023). As such, this compound displays potential to overcome part of the cause of acquired resistance.

Whilst adagrasib and sotorasib are similar, the mutational burden which confers resistance to their treatment differs (Koga et al., 2021). G13D, R68M, A59S and A59T mutations can render sotorasib ineffective, whereas tumours with these mutations remain sensitive to adagrasib. Alternatively, mutations at M72 or Q99 confer adagrasib resistance but do not affect sotorasib sensitivity, meaning that potentially the use of both drugs after the emergence of resistance to a single inhibitor has occurred may benefit patients (Koga et al., 2021). Furthermore, mutations at H95 can also render adagrasib ineffective, since this drug relies on the presence of H95 to sufficiently bind and elicit its effects (Mahran et al., 2024). In this way, genotyping and stratifying patients for mutation-specific inhibitors proves essential for improving patient outcomes.

Modifications causing activation of receptor tyrosine kinase/Ras signalling pathway cascades, independent of direct KRAS mutations, can also cause acquired resistance to Ras targeting therapies (Awad et al., 2021). Across the cohort of resistant patients studied, 18% had multiple overlapping genetic causes for this. These patients harboured high-level amplification of the KRAS G12C allele, developed bypass resistance mechanisms such as MET amplification, suffered oncogenic fusions, loss of function mutations and acquired activating mutations in proteins such as NRAS, NF1, and B-Raf (Kwan et al., 2022; Suzuki et al., 2021). Histological transformation of lung adenocarcinoma to squamous cell carcinoma was observed in some patients, occurring independently of other resistance mechanisms, though the cause for this is, as yet, unknown (Awad et al., 2021). This study echoes the complexity of resistance to Ras-targeting therapies, and, similarly to any other drug, resistance will occur and create further therapeutic barriers once these are in common clinical use (Kwan et al., 2022).

Furthermore, addition of Ras-effector inhibitor therapies are believed to enhance the effects of tipifarnib, through reduction of compensatory MAPK/PI3K pathway signalling potentially brought about by HRAS inhibition in head and neck cancer cell lines (Javaid et al., 2022). Instead, combined treatment with inhibitors of these pathways appeared to elicit apoptosis, with ERK inhibition also reversing the epithelial-to-mesenchymal transition, which can be stimulated by tipifarnib (Javaid et al., 2022).

*In vitro* evidence suggests development of adaptive resistance to the KRAS-G12C inhibitor ARS-1620, the structural precursor to AMG510, in an NSCLC cell line was due to a subpopulation of cells synthesizing new KRAS-G12C proteins, corresponding with increased EGFR signalling and aurora kinase A activation (Xue et al., 2020). This signalling enables any newly expressed KRAS G12C protein to remain in an active GTP-bound state, causing alterations to the cell cycle and ultimately eliciting a constant pro-proliferative effect (Xue et al., 2020).

The involvement of the aurora kinase pathway indicates that there is a need to perhaps shift direction in the treatment of commonly associated Ras-mediated kinase pathways (e.g. MAPK/PI3K-AKT), and instead consider a more individualised approach to Ras-mediated disease. Phase I clinical trials are currently recruiting to examine the effect of aurora kinase inhibitor LY3295688 in KRAS G12C mutant solid tumours (NCT04956640) (Ammakkanavar et al., 2022). Furthermore, combination therapy of VIC-1911, another aurora kinase inhibitor, with sotorasib, was initiated in a Phase I clinical trial, although recruitment has since been terminated at the sponsor’s decision (NCT05374538) (Goldberg et al., 2023).

Additional proposed mechanisms by which resistance may occur to direct Ras inhibitors include feedback mechanisms within Ras-mediated pathways. Such concept is already seen amongst resistance to inhibitors targeting other elements of the Ras pathway, including that to EGFR inhibition (Ercan et al., 2012). Downregulation of negative regulators of ERK has also been reported to cause the reactivation of ERK (Ercan et al., 2012; Bruner et al., 2017). Furthermore, studies have indicated a need to inhibit multiple members of the MAPK pathway in KRAS-mutated PDAC, such as Raf and ERK, to fully inhibit *MYC* transcription and oncogenic signalling. This also caused decrease in other activated kinase pathways, including the aurora kinase pathway (Ozkan-Dagliyan et al., 2020). This would allow for continued oncogenic Ras-associated signalling despite Ras therapeutic targeting, deeming the cancer cells able to continue to proliferate and harbour resistance to apoptosis.

Avutometinib is a newer MEK inhibitor and has shown some early promise in early Phase I trials and is being trialled as a monotherapy and alongside adagrasib and sotorasib as a means of increasing inhibition throughout the MAPK pathway. Patients enrolled on the monotherapy trial have a spectrum of KRAS and Ras-mediated pathway mutations, including HRAS G13R, KRAS G12V, KRAS G12R, KRAS G12D, and BRAF V600E (Poh, 2021). Initial results exhibited promise, with further trials now also active and recruiting (NCT02407509, NCT06104488, NCT05074810, NCT05200442, NCT05375994) (Guo et al., 2020). This is promising with regards to the minimisation of the impact of secondary mutations which may occur in the Ras pathway in response to KRAS G12C inhibitors, as indicated in analysis of adagrasib-resistant patients (Awad et al., 2021).

Other studies have shown potential for an increase in wild-type Ras signalling within the cell as a means of re-inducing proliferative signalling, to effectively bypass oncogenic KRAS and its mutant-specific inhibition (Ryan et al., 2022). This could however be overcome using SHP2 inhibitors, which have previously been shown to mediate the effects of drug resistance mechanisms in Ras-mutant breast cancer, PDAC and lung cancer models (Ahmed et al., 2019; Fedele et al., 2020; Ryan et al., 2022; Sealover et al., 2024). In the case of KRAS G12C inhibitor therapy, SHP2 inhibition in combination with KRAS G12C inhibition, using SHP099 and ARS-1620, respectively, was shown to confer alterations to the tumour microenvironment and a decrease in MAPK pathway activation, thereby inhibiting tumour growth (Fedele et al., 2020). This is accompanied by inhibition of tumour regrowth of adagrasib-resistant colorectal tumours in cell line-derived xenograft mice, when they were co-treated with the SHP2 inhibitor TNO155 (Thatikonda et al., 2023). Co-inhibition of SHP2 and KRAS G12C is now being explored clinically, including in the HERKULES-2 clinical trial (NCT04959981 and NCT04330664). SHP2 inhibition may also reduce incidence of resistance to other Ras-pathway targeting therapeutics, SHP2 inhibition seen to ameliorate resistance to osimertinib, the EGFR inhibitor, in lung adenocarcinoma cell lines (Sealover et al., 2024). This builds on previous studies, which indicated sensitivity to the EGFR inhibitor gefitinib also relies on SHP2 expression (Lazzara et al., 2010). SHP2 inhibition using TNO155 was also investigated in conjunction with JDQ443, with 33% of patients exhibiting confirmed responses, and a disease control rate of 67%, accompanied by a tolerable safety profile (NCT04699188) (Negrao et al., 2023b).

Use of a SOS1 inhibitor could also help mediate KRAS G12C inhibitor resistance, such as using novel compound BI-3406 (Thatikonda et al., 2023). *In vitro*, there was decreased outgrowth of adagrasib-resistant cell lines treated with the two inhibitors, compared to those treated with purely adagrasib, thereby indicating a slower proliferative potential and overcoming of the resistant phenotype (Thatikonda et al., 2023). Co-inhibition of SOS1 and KRAS G12C was found to alter the MAPK transcriptome, and exhibit synergy in inhibiting ERK phosphorylation (Daley et al., 2023b). This was accompanied by evidence of decreased tumour volume in cell line derived colorectal cancer mouse models. This data was sufficient in launching clinical trials studying the combined inhibition of SOS1 and KRAS G12C using BI1701963, a derivative of BI-3406 (NCT04185883 and NCT04973163) (Thatikonda et al., 2023). Expansion of the KRYSTAL trial repertoire also included evaluation of BI1701963, compared to adagrasib (KRYSTAL-14, NCT04975256).

Aside from canonical Ras/receptor tyrosine kinase signalling pathways, it has been recently reported that the regulation of cellular oxygen may be involved in mediating KRAS G12C inhibitor resistance. Patients with mutations in the *KEAP1* gene, which is considered a tumour suppressor gene and responsible for regulation of reactive oxygen species in the cell, appear to confer poorer outcomes (Negrao et al., 2023a). Furthermore, activation of hypoxic response was also shown to be dysregulated in KRAS G12C inhibitor tolerant persister cells, though the effects of this could largely be abrogated by SOS1 inhibition (Daley et al., 2023a).

Involvement of the Hippo signalling pathway, including YAP/TAZ-TEAD activation, which is frequently over-expressed in cancer (Baroja et al., 2024) also appeared to confer resistance to adagrasib. This was seen to be Rho-dependent, which is another small GTPase, which can cross-talk with Ras to instigate proliferative signalling (Sahai et al., 2001) with inhibition of this pro-proliferative and pro-tumoral pathway using siRNA or appearing to increase sensitivity to adagrasib (Mukhopadhyay et al., 2023). This can be achieved using TEAD inhibitors, which overcame the increased transcription of oncogenes such as *MYC*, as well as inducing apoptosis, which was inhibited by aberrant PI3K/AKT pathway activity, stimulated by TEAD in KRAS G12C inhibitor resistant cell lines (Edwards et al., 2023).

## Conclusion

Overall, resistance to direct Ras inhibitors is highly diverse and heterogeneous, with potential for multiple mechanisms to act within one patient. There are, however, potential methods that could reduce the risk of resistance occurring, predominantly the use of combination therapies. Such concept is currently being explored within newer remits of the CodeBREAK and KRYSTAL trials, through combination with anti-EGFR therapy, as well as with MEK inhibition, using trametinib or avutometinib (NCT04185883, NCT05074810, NCT05375994) (Supplementary Table S2).

This review has highlighted the importance of understanding the intricacies of Ras mutant signalling and explored the differences in kinase activation in the presence of these mutants. The numerous kinases and signalling pathways activated by Ras mean that there is a wealth of targets possible in the treatment of Ras-mutant cancer, though this must be taken with caution. Instead, the overall mutational status of each cancer must be carefully considered, to select the best pharmacological therapy for each individual patient, which may be Ras-targeting, or downstream kinase targeting instead. Given the significant enhancement of personalised medicine in recent years, as well as the game-changing developments of Ras-mutant-specific inhibitors, the future of Ras therapeutics, and the patient benefit these will bring, is highly promising.
